# Functional Trait Space and Multiscale Allometric Scaling of Different Architectural Types in *Malus*

**DOI:** 10.3390/plants15091347

**Published:** 2026-04-28

**Authors:** Yuerong Fan, Yiting Shen, Ruomiao Zhou, Wangxiang Zhang

**Affiliations:** 1 College of Forestry, Nanjing Forestry University, Nanjing 210037, China; fyr@njfu.edu.cn (Y.F.);; 2 Co-Innovation Center for Sustainable Forestry in Southern China, Nanjing Forestry University, Nanjing 210037, China

**Keywords:** *Malus*, plant architecture, functional traits, multidimensional functional space, allometric scaling, functional diversity, resource allocation

## Abstract

Tree architecture is a critical determinant of plant performance, light capture, biomechanical stability, and resource allocation. However, the multidimensional functional trait space and multiscale allometric scaling mechanisms underlying different architectural types in *Malus* remain poorly understood. This study investigates the multidimensional functional trait space and multiscale allometric scaling relationships among three typical architectural types (weeping, upright, and spreading) in *Malus*. A total of 206 germplasm accessions were analyzed by integrating nine core functional traits spanning macro-architectural, branch biomechanical, and leaf economic dimensions. Principal component analysis revealed that architectural differentiation is primarily driven by macro-architectural and branch biomechanical traits, alongside coordinated contributions from leaf economic traits. Functional diversity analysis indicated that the upright and spreading types exhibited higher functional richness, while the weeping type displayed the highest functional divergence but minimal or no functional overlap with the upright and spreading type, reflecting strong niche specialization under artificial selection. Multiscale allometric analyses demonstrated significant divergence in resource allocation strategies across hierarchical levels. At the whole-tree level, architectural types differed markedly in height–diameter and height–crown scaling relationships. At the branch level, conserved positive allometric scaling was observed, with the weeping type showing higher intercepts indicative of increased mechanical investment. At the leaf level, consistent negative allometry between petiole length and leaf area suggested optimized resource allocation for light capture. These pronounced differences suggest distinct ecological adaptation strategies: the weeping type prioritizes biomechanical compensation for pendulous branches and optimized light capture in loose canopies; the upright type emphasizes vertical light competition and mechanical compactness; the spreading type balances lateral expansion and spatial filling efficiency, reflecting differentiated resource allocation patterns shaped by artificial selection. Overall, this study reveals that tree architecture in *Malus* is shaped by coordinated trait interactions across multiple scales, leading to distinct ecological strategies and resource allocation patterns. These findings provide new insights into the structure–function co-evolution of woody plants and offer a theoretical framework for functional trait-assisted breeding of ornamental tree architectures.

## 1. Introduction

Plant architecture is the outcome of interactions between genetic regulation and environmental stresses. It not only determines a plant’s spatial occupation capacity but also profoundly influences its light interception efficiency, biomechanical stability, and resource allocation strategies [1]. An optimal plant architecture confers significant advantages in several key aspects, including the capture of photosynthetically active radiation, mechanical resistance to wind loads, and overall survival and reproductive success, thereby promoting efficient biomass accumulation and enhancing adaptability to variable environmental conditions [2]. Therefore, a deep understanding of the patterns, formation processes, and influencing factors of plant architecture holds substantial importance for the breeding of superior varieties and the sustainable management of forestry resources [3].

In recent years, the rise of functional traits research has provided powerful tools for quantitatively dissecting the relationships between plant structure and function [4,5]. Functional traits are defined as morphological, physiological, biochemical, structural, phenological, or behavioral characteristics that influence individual performance, fitness, and niche occupancy [6]. These traits have been widely applied in fields such as community assembly mechanisms [7], species invasion processes [8], and adaptation to global climate change [9]; however, a unified consensus remains lacking in the field of ornamental horticulture. Díaz et al. [10], based on a global dataset of plant form and function, proposed the “Global Spectrum of Plant Form and Function,” which compresses approximately three-quarters of plant trait variation into two primary dimensions. The first dimension reflects the size of whole plants and their organs, embodying the balance between mechanical support for tree architecture and water transport in branches [11,12]. The second dimension corresponds to the Leaf Economics Spectrum (LES), which captures trade-offs among leaf area, petiole investment, carbon assimilation rate, nutrient content, and leaf lifespan [13,14]. The synergies and trade-offs among multidimensional functional traits under evolutionary and selective pressures collectively define the functional trait space. The functional trait space is a multidimensional phenotypic hypervolume delineated by axes of key plant functional traits [15]. Building on this space, indices of functional diversity (such as functional richness and functional divergence) enable quantitative characterization of the breadth of niche occupancy by species or functional groups, as well as the degree of differentiation in resource-use strategies [16]. Therefore, integrating organ-level (micro-scale) traits—such as those of leaves and branches—with whole-tree (macro-scale) architectural traits into a multidimensional functional space from a functional trait perspective facilitates quantitative description of phenotypic distribution patterns in plants. It also helps reveal the intrinsic decision-making logic underlying resource acquisition versus structural construction, along with the adaptive mechanisms supporting specific survival strategies [17].

However, few studies have integrated macro-architectural, branch biomechanical, and leaf economic traits into a unified multidimensional functional trait space to quantitatively dissect resource allocation trade-offs across hierarchical scales, especially in ornamental woody species shaped by artificial selection. Key gaps remain in understanding how distinct architectural types (weeping, upright, spreading) in *Malus* diverge in functional niches and allometric strategies.

Allometric scaling refers to the power-law relationships between sizes of different organs or traits, reflecting resource allocation strategies [18]. According to Metabolic Scaling Theory (MST), growth relationships between different plant organs typically follow power-law functions [19]. Studies have demonstrated that, across a vast size range spanning over 20 orders of magnitude—from microscopic unicellular algae and aquatic ferns to macroscopic terrestrial herbaceous and woody plants—highly conserved allometric scaling exponents are observed, which maintain fundamental biomechanical stability and resource transport efficiency [20]. Nevertheless, specific environmental stresses and artificial selection can substantially drive diversification in plant architecture by altering scaling exponents or intercepts. For instance, in mature tree communities within tropical rainforests, allometric relationships between leaves and branches deviate markedly from those in seedlings to balance intense spatial competition with the substantial mechanical costs of trunk support [21]. Similarly, during the domestication of crops such as sunflower, tomato, and cabbage, human selection has significantly elevated the intercept for allocation to leaves and other specific organs relative to whole-plant biomass, resulting in larger overall size and markedly distinct morphological architectures compared to their wild progenitors [22]. In essence, whether shaped by natural environmental forces or human-directed breeding interventions, this diversification in tree architecture ultimately manifests as resource reallocation across multiple structural hierarchical scales. At the whole-tree level, the allometric trade-off between tree height and basal diameter determines mechanical stability and vertical competitive ability [23]. At the branch level, the allometric relationship between branch length and basal diameter adheres to Corner’s rule, which elucidates the balance between mechanical support costs and spatial expansion efficiency [24,25]. At the leaf level, the negative allometric scaling between petiole dimensions and leaf area reflects fine-tuned optimization of support costs for photosynthetic organs [26,27]. Dissecting differences in allometric scaling across these multiple scales not only enables quantitative revelation of the multi-level resource allocation rules in plants but also provides a theoretical foundation for directional breeding of tree architecture and the development of early-stage selection models based on functional traits [28].

*Malus* spp. (crabapple), as an important ornamental tree within the genus *Malus* of the Rosaceae family, exhibits exceptionally high genetic diversity and phenotypic variability [29,30]. Through long-term natural evolution and artificial selection, crabapples have developed three typical architectural types: weeping type (WT), upright type (UT), and spreading type (ST) [31]. These types display pronounced differences in tree size, branching angles, and leaf morphology [32], making them an ideal model for investigating how tree architecture drives the remodeling of functional traits and multi-level allometric trade-offs. However, existing studies have largely been limited to qualitative classification and evaluation of tree architectures [33] or focused on the genetic basis of single traits (e.g., columnar [34] or weeping [35,36]). From a functional ecology perspective, leveraging a multidimensional trait space offers a promising approach for quantitatively analyzing resource allocation and trade-off mechanisms across different tree architectures at the levels of whole-plant morphology, branch biomechanics, and leaf economics.

Based on the observed differences in tree height, branching angles, and leaf morphology among the three architectural types, we hypothesize that: (1) the WT will exhibit specialized functional traits favoring vertical extension and biomechanical reinforcement to compensate for gravity-induced drooping; (2) the UT will show traits optimized for mechanical compactness and efficient light capture within a narrow canopy; (3) these differences will result in divergent resource allocation patterns manifested as distinct allometric scaling relationships at the whole-tree, branch, and leaf levels. The corresponding null hypotheses assume no significant differences in multidimensional functional trait space occupancy, functional diversity indices, or multiscale allometric scaling relationships among the three architectural types. These null hypotheses were statistically tested using one-way ANOVA with Tukey HSD post hoc tests, kernel density estimation with functional diversity metrics, and standardized major axis (SMA) regression with common slope tests.

Given this background, the present study utilized 206 germplasm accessions from the National Crabapple Germplasm Repository of China (encompassing the three typical architectural types: weeping type, upright type, and spreading type). It integrated core functional traits across scales, from macro-level whole-tree architecture to micro-level leaf traits, with the following objectives: (1) to elucidate the phenotypic distribution patterns of different tree architectures within the multidimensional functional trait space and to identify the primary functional dimensions driving architectural variation; (2) to evaluate the differentiation in functional diversity among tree architectures and to reveal the impacts of artificial selection pressures on niche occupancy and evolutionary strategies in *Malus*; and (3) to dissect cross-hierarchical allometric relationships in *Malus* and to clarify the biomechanical trade-offs and resource allocation logic underlying architectural specialization. This study not only provides theoretical support for directional breeding of tree architecture in woody ornamental plants but also contributes novel ecological insights into the co-evolutionary dynamics of structure and function in perennial woody species.

## 2. Results

### 2.1. Variations in Functional Traits Across Different Architectural Types

**Figure 1:**
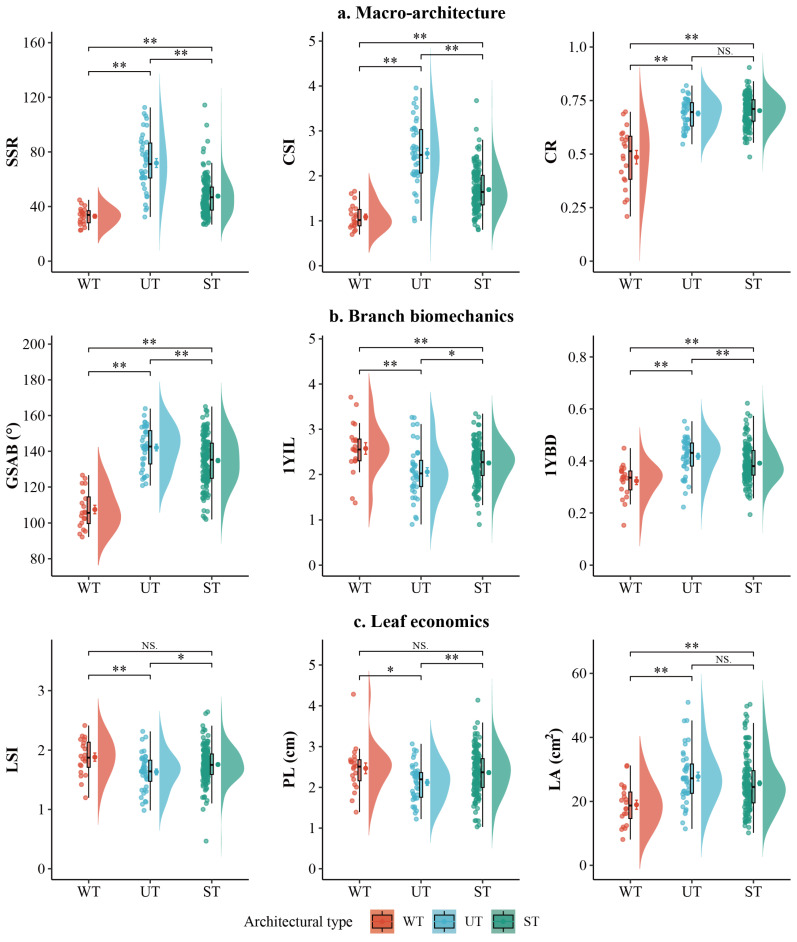
Variation of nine core functional traits across three architectural types in *Malus*. Traits are grouped into three functional categories: (**a**) macro-architecture, (**b**) branch biomechanics, and (**c**) leaf economics. Raincloud plots combine half-violin (density), boxplot (median and IQR), and jittered raw points. Dots with error bars represent mean ± standard error (SE). Asterisks indicate significant differences (* *p* < 0.05, ** *p* < 0.01; one-way ANOVA followed by Tukey HSD post hoc test). Colors correspond to architectural types: WT (weeping type, red), UT (upright type, blue), ST (spreading type, green).

The nine core functional traits of the three *Malus* architectural types exhibited significant variations across macro-architecture, branch biomechanics, and leaf economics (Figure 1).

Regarding macro-architecture (Figure 1a), the UT showed clear dominance in the vertical dimension, with its mean stem slenderness ratio (SSR) and crown shape index (CSI) being significantly higher than those of the other two types (*p* < 0.01). Conversely, the WT showed significantly lower mean values for all three macro-architectural traits compared to the other types (*p* < 0.01). The crown ratio (CR) of WT was significantly lower than that of both UT and the ST (*p* < 0.01), while no significant difference was observed between UT and ST.

In terms of branch biomechanics (Figure 1b), WT exhibited significantly lower mean values for the basal gravitropic set-point angle of the main branch (GSAB) and 1-year-old shoot basal diameter (1YBD) than UT and ST (*p* < 0.01), whereas its mean internode length on 1-year-old shoots (1YIL) was significantly higher than the others (*p* < 0.01). UT possessed the thickest 1YBD and the largest GSAB (*p* < 0.01), while the three traits of ST occupied an intermediate position between the two.

In the aspect of leaf economics (Figure 1c), UT was characterized by the lowest mean leaf shape index (LSI), the shortest petiole length (PL), and the largest leaf area (LA) (*p* < 0.01), suggesting a strategy of larger, more orbicular leaves with a compact spatial arrangement. In contrast, WT displayed the highest mean LSI, the longest PL, and the smallest LA, indicating smaller, more elongated leaves with a loose arrangement. For ST, the LSI and PL showed no significant differences from WT, while its LA was comparable to that of UT.

### 2.2. Functional Trait Space and Principal Component Analysis (PCA)

**Figure 2:**
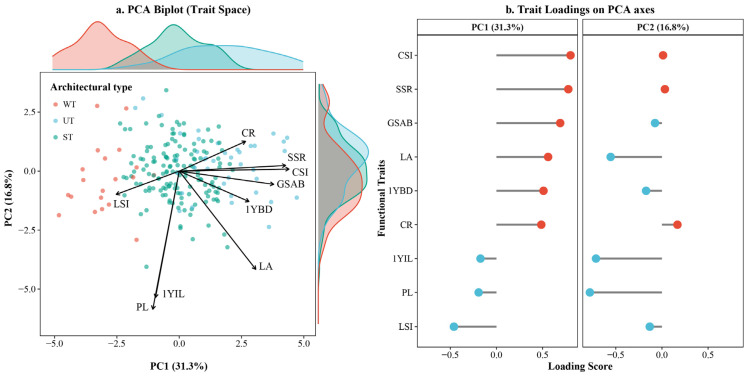
Principal component analysis (PCA) of the nine core functional traits across three architectural types in *Malus*. (**a**) PCA biplot showing the distribution of the weeping type (WT), upright type (UT), and spreading type (ST) in functional space; marginal density plots display distributions along PC1 and PC2; arrows indicate direction and magnitude of functional trait vectors. (**b**) Lollipop plot of trait loadings on PCA axes (red and blue dots represent positive and negative loadings, respectively). Colors correspond to architectural types: WT (red), UT (blue), ST (green).

To further explore the synergistic relationships among functional traits and construct a trait space, a Principal Component Analysis (PCA) was performed based on the nine core functional traits. The first two principal components accounted for 48.1% of the total phenotypic variance, with PC1 and PC2 explaining 31.3% and 16.8% of the variation, respectively. The PCA biplot (Figure 2a) revealed distinct distribution patterns among the three architectural types. The WT was clearly segregated from the other two types, predominantly occupying the negative region of the PC1 axis, whereas the UT and ST exhibited substantial overlap in the positive region of PC1. Specifically, WT showed a tendency to cluster toward lower values along the PC1 axis, while UT tended to aggregate toward higher values along the PC2 axis.

Analysis of trait loadings identified the primary drivers shaping the trait space (Figure 2b). PC1 was primarily driven by positive loadings from macro-architectural and branch biomechanical traits, showing exceptionally high positive scores for crown shape index (CSI), stem slenderness ratio (SSR), and basal gravitropic set-point angle of the main branch (GSAB). In contrast, the leaf shape index (LSI) exerted a strong negative loading on PC1. PC2 was mainly influenced by negative loadings from petiole length (PL), internode length on 1-year-old shoots (1YIL), and leaf area (LA), reflecting differences in plant spatial extension and light interception area. These loading patterns indicate that the architectural types of *Malus* are not driven by a single trait but are collectively determined by a suite of traits across multiple functional categories.

### 2.3. Functional Diversity Indices and Overlap in Multidimensional Functional Space

**Figure 3:**
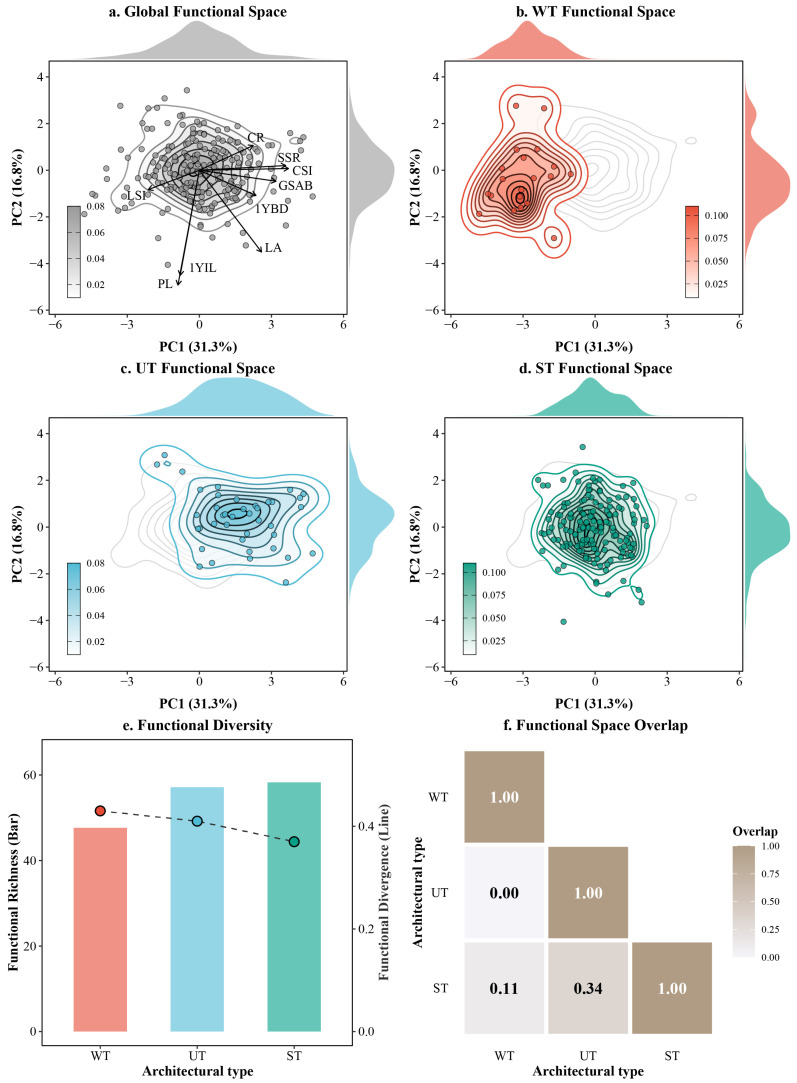
Functional space occupation, diversity, and overlap among the three architectural types in *Malus*. (**a**) Global 2D functional space with superimposed functional trait vectors. (**b**–**d**) 2D kernel density contour plots showing the specific spatial distribution of the weeping type (WT), upright type (UT), and spreading type (ST) within the global PCA space; color gradients represent probability density of accessions (darker shades indicate higher data concentration). (**e**) Functional diversity indices for the three architectural types: bar chart (left y-axis) shows Functional Richness (FRic), line-and-scatter plot (right y-axis) shows Functional Divergence (FDiv). (**f**) Pairwise functional space overlap matrix among architectural types (values indicate proportion of shared functional space). Colors correspond to architectural types: WT (red), UT (blue), ST (green).

The mapping of the multidimensional functional space via two-dimensional kernel density estimation (Figure 3a–d) revealed distinct occupancy patterns among the three *Malus* architectural types. The ST and UT occupied broader regions within the global functional space, with functional richness (FRic) values of 58.26 and 57.15, respectively (Figure 3e). This larger functional volume suggests that ST and UT encompass a wider range of phenotypic variations, enabling them to adapt to more diverse spatial resources and light interception strategies. In contrast, the WT was confined to a narrower and highly specialized region on the left side of the functional space (Figure 3b), exhibiting the lowest FRic value (47.65) (Figure 3e).

Notably, despite its constrained functional volume, WT exhibited the highest functional divergence (FDiv = 0.43) (Figure 3e). This indicates that individuals within this group tend to be distributed toward the extreme edges of their specific phenotypic niche rather than clustering near the center. Furthermore, the pairwise functional overlap (FOve) analysis (Figure 3f) quantitatively confirmed the strict phenotypic isolation of WT, which showed zero overlap (0.00) with UT and extremely low overlap (0.11) with ST. Conversely, UT and ST exhibited a substantial overlap (0.34), suggesting that despite their visual differences in tree structure and branching angles, they share considerable similarities in their ecological strategies across macro-architecture, branch biomechanics, and leaf economics.

### 2.4. Multiscale Allometric Scaling Relationships

**Figure 4:**
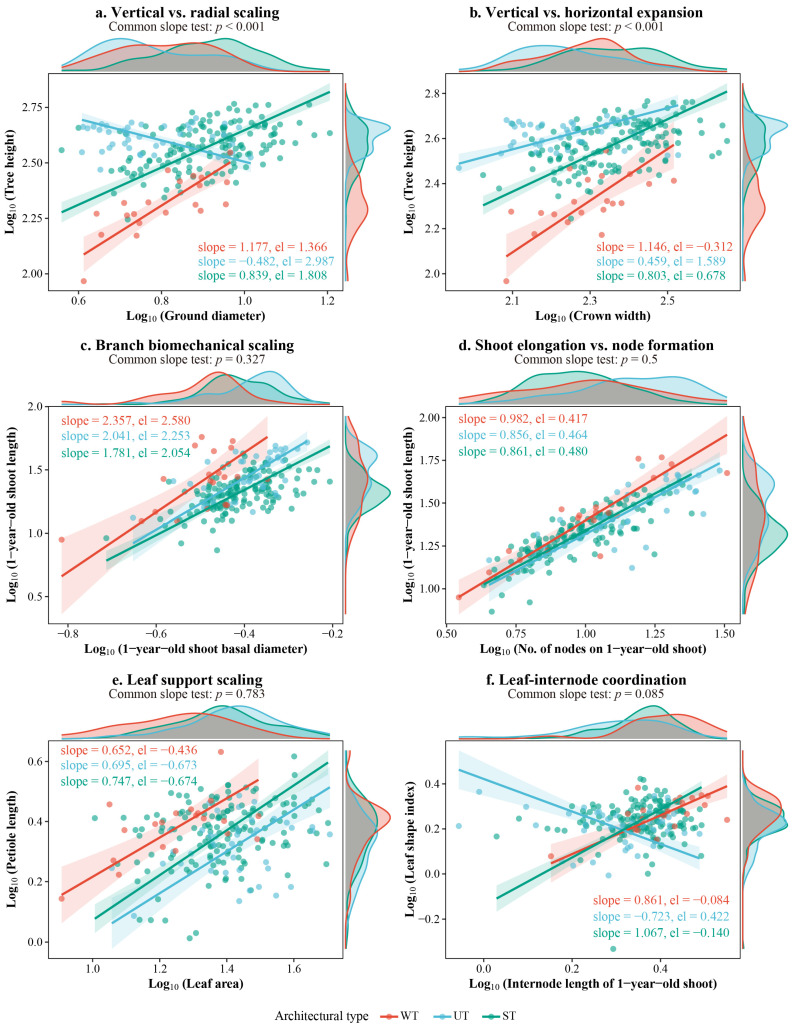
Standardized Major Axis (SMA) regression of multiscale allometric scaling relationships across three architectural types of *Malus*. (**a**,**b**) Whole-tree level; (**c**,**d**) branch level; (**e**,**f**) leaf level. SMA regression lines and their 95% confidence bands (shaded areas) are shown for each architectural type (WT: weeping type, red; UT: upright type, blue; ST: spreading type, green). Slope and intercept (*el*) values are annotated in each panel, along with common slope test *p*-values. Significant results (*p* < 0.05) indicate divergence in allometric slopes, while non-significant results (*p* > 0.05) suggest a common scaling exponent among groups. Biologically, significant slope divergences (*p* < 0.05) at the whole-tree level (**a**,**b**) highlight distinct strategic trade-offs between mechanical stability and spatial expansion. Shared common slopes (*p* > 0.05) at the branch (**c**,**d**) and leaf (**e**,**f**) levels reveal highly conserved developmental trajectories, where further significant differences in intercepts (*el*) reflect group-specific structural over-investments (e.g., enhanced radial support in WT) or fine-tuned adaptations for light capture.

To evaluate the allometric trade-off strategies in resource allocation and structural assembly among different *Malus* architectural types, Standardized Major Axis (SMA) regression was performed across three hierarchical levels (Figure 4). The common slope test was used to determine whether the scaling exponents (slopes) differed significantly among the three architectural types. A non-significant common slope test (*p* > 0.05) indicates that the three types share a common scaling exponent, suggesting conserved biomechanical or physiological constraints, whereas a significant result (*p* < 0.05) reveals divergent allometric strategies shaped by architectural specialization.

(1) Whole-tree level: Allometric scaling at the whole-tree level reflects the equilibrium between vertical extension, radial support, and horizontal expansion. Vertical vs. radial scaling (TH vs. GD, Figure 4a): Significant divergence in slopes was observed among the three types (*p* < 0.001). The UT exhibited a unique negative allometric trend (slope = −0.482), reflecting a strong structural constraint between height increment and radial support, or a tendency to maintain height once a certain developmental stage is reached. In contrast, the WT showed strong positive allometry (slope = 1.177), where height increased rapidly with increasing diameter. Vertical vs. horizontal expansion (TH vs. CW, Figure 4b): The relationship between height and crown width also showed significant slope divergence (*p* < 0.001). WT exhibited the steepest slope (slope = 1.146), indicating that height growth outpaced crown expansion. This aligns with the characteristic of restricted crown width due to drooping primary branches and high vertical variability. Conversely, the slope of UT was minimal (slope = 0.459), reflecting its narrow canopy and constrained height growth.

(2) Branch level: Synergistic relationships at the branch level reveal the developmental patterns of 1-year-old shoots. Branch biomechanical scaling (1YSL vs. 1YBD, Figure 4c): The three types followed a highly consistent positive allometric pattern (common slope *p* = 0.327), with all slopes significantly greater than 1 (1.781–2.357). This suggests that shoot elongation depends on a disproportionate increase in basal diameter to maintain mechanical stability. Notably, WT exhibited the highest intercept (*el* = 2.580), meaning that for a given shoot length, WT allocates the most to basal support. This “over-investment” in radial support may compensate for the higher bending moments caused by its gravity-driven drooping habit. Shoot elongation vs. node formation (1YSL vs. 1YSN, Figure 4d): All types shared a common slope (*p* = 0.5) near 1, indicating isometric scaling where node formation is coupled with shoot elongation. However, ST possessed the highest intercept (*el* = 0.480), suggesting superior meristematic activity and spatial-filling capacity per unit shoot length. In contrast, WT had the lowest intercept (*el* = 0.417), reflecting the most diffuse arrangement of nodes.

(3) Leaf level: The interaction between leaves and shoots reflects the fine-scale economic strategies of different tree architectural types. Leaf support scaling (PL vs. LA, Figure 4e): A consistent allometric pattern was observed across types (*p* = 0.783), with all slopes less than 1 (0.652–0.747). This indicates that as leaf area increases, the proportional investment in petiole length decreases, optimizing resource use in leaf construction. Leaf-internode coordination (LSI vs. 1YIL, Figure 4f): The common slope test yielded *p* = 0.085, indicating that while slope divergence did not reach extreme significance, distinct evolutionary trends were evident. UT was the only type showing a negative allometric tendency (slope = −0.723), where leaves became more orbicular (lower LSI) as internode length increased, likely an adaptation to optimize light interception on rapidly elongating shoots. In contrast, both WT (slope = 0.861) and ST (slope = 1.067) exhibited positive allometry, where leaves became more elongated as internodes lengthened. ST showed the steepest slope, suggesting that its leaf morphology is most sensitive to spatial expansion.

## 3. Discussion

### 3.1. Phenotypic Distribution Patterns and Driving Dimensions of Functional Trait Space Across Different Architectural Types in Malus

In this study, principal component analysis (PCA) was applied for the first time to integrate micro-scale organ traits with macro-scale tree architecture across 206 *Malus* germplasm accessions, quantitatively revealing significant differences in trait space occupancy among WT, UT, and ST *Malus*: WT predominantly occupied the negative half of PC1 and was clearly separated from the other two types, whereas UT and ST showed substantial overlap along the positive half of PC1 (Figure 2a). PC1 exhibited high loadings reflecting a trade-off between vertical light competition and mechanical stability, while PC2 captured optimization strategies for spatial extension and light interception (Figure 2b). These findings align closely with Maynard et al. [37] regarding global relationships in tree functional traits. The results confirm that variation in *Malus* tree architecture is primarily driven positively by macro-architectural and branch biomechanical traits (SSR, GSAB, CSI), with secondary contributions from leaf economics traits (LSI, PL, LA). These findings are highly consistent with recent global studies on functional trait spaces and multiscale allometric scaling in woody plants [38,39].

The specialized separation of WT along the negative end of PC1 reflects its low CSI resulting from reduced basal gravitropic set-point angle of the main branch (GSAB) at the base of primary branches, combined with a loose leaf arrangement pattern. From a molecular perspective, Furutani et al. [40] demonstrated that gravitropic set-point angle is primarily regulated by polar auxin transport, with the *LAZY* gene family playing a pivotal role. *LAZY* proteins modulate the asymmetric distribution of auxin transport proteins (PINs) on the plasma membrane, generating differential auxin concentrations across branch sides and thereby altering growth direction. The overlap between UT and ST suggests similarity in their resource acquisition niches. Coupel-Ledru et al. [41] identified independent genetic control mechanisms for tree architecture and light-capture functional traits in apple core germplasm, while Hollender and Dardick [42] elucidated molecular mechanisms showing that genetic and hormonal regulation jointly drive branch spatial extension patterns.

Thus, by quantitatively positioning core functional traits, this study delineates the ecological niches of *Malus* and further substantiates that tree architecture is not dominated by single traits but results from synergistic interactions across multiple functional categories (macro-architecture, branch biomechanics, and leaf economics). These findings provide a novel functional ecology perspective on traditional qualitative classifications and lay a theoretical foundation for early phenotypic screening in directional breeding programs.

From a functional trait perspective, the three architectural types exhibit distinct environmental adaptation and resource utilization strategies shaped by coordinated trait syndromes [43]. The WT achieves biomechanical compensation for pendulous branches through low SSR and CSI, longer internodes (1YIL), smaller narrower leaves (low LA, high LSI), and longer petioles (PL), coupled with higher branch allometric intercepts that enhance radial support and reduce self-shading in loose canopies, favoring light capture and vertical extension in open or shaded microenvironments. In contrast, the UT prioritizes vertical light competition and mechanical compactness via high SSR, CSI, thick basal diameters, large orbicular leaves, and short petioles, resulting in conservative height–diameter allometry and efficient carbon assimilation in high-light, dense-planting conditions. The ST balances lateral expansion with dense node production and flexible leaf morphology, conferring higher functional richness and broader niche breadth for adaptation to heterogeneous light environments. These differentiated trait combinations illustrate how artificial selection has sculpted specialized versus generalist resource allocation strategies in *Malus*.

### 3.2. Functional Diversity Characteristics of Different Architectural Types in Malus and the Influence of Artificial Selection

In this study, two-dimensional kernel density estimation combined with functional diversity indices was employed for the first time in *Malus* to quantitatively assess functional richness, functional divergence, and functional overlap. The results revealed that the ST and UT exhibited significantly higher functional richness (FRic) than the WT (58.26 and 57.15 versus 47.65, respectively), whereas WT displayed the highest functional divergence (FDiv = 0.43) and extremely low functional space overlap with both UT and ST (0.00 and 0.11, respectively) (Figure 3). It is noteworthy that sample sizes were markedly unbalanced across the three architectural types (only 20 accessions for the WT versus 146 for the ST; see Table 1). This imbalance may lead to underestimation of functional richness (FRic) in smaller sample groups (e.g., WT). However, kernel density estimation methods are relatively robust to uneven sampling, and our use of two-dimensional PCA projections further mitigates sample-size bias [44]. These findings indicate that artificial selection has shaped differentiated ecological strategies among *Malus* architectural types: ST and UT have inherited and expanded broader functional ecological niches, occupying larger trait space volumes and thereby conferring greater potential adaptability to variable environments (such as light-resource gradients and spatial competition). In contrast, WT has become highly specialized under long-term directional selection; although its functional space is compressed, it exhibits extreme divergence at the margins of its specific niche, reflecting a pronounced trade-off between niche specialization and functional extremization.

The elevated FRic of ST and UT likely stems from the rich genetic backgrounds of North American and self-bred cultivars (Table 1: North American cultivars comprise 72 spreading-type accessions, while self-bred cultivars include 59 spreading-type accessions). These sources integrate a wider range of wild ancestral traits and hybrid variation, thereby maintaining higher overall functional breadth. Conversely, the low FRic combined with high FDiv in WT reflects the reinforcement of pendulous branching under intense artificial directional selection, resulting in highly conserved trait syndromes [43] and spatial compression. Nevertheless, the dispersed distribution of extreme individuals at the functional space margins enhances ecological specificity in particular microhabitats (e.g., weeping garden landscapes, shaded sites, or wind-resistant positions). This pattern is consistent with the findings of Cantarel et al. [45] in wheat cultivars, where artificial selection typically compresses the overall volume of functional trait space but preserves or intensifies extreme variation for targeted traits, leading to the coexistence of conserved trait syndromes and specialized trajectories.

Unlike Cantarel et al. [45], who focused on functional variation between crop cultivars and wild progenitors, the present study further quantifies functional space differentiation among distinct architectural groups within the same genus. The results demonstrate that artificial selection has not diminished overall ecological-strategy diversity; rather, through architecture-specific selection, it has promoted clear phenotypic niche partitioning and functional complementarity. This differentiation offers novel insights for the functional ecology of ornamental woody plants: artificial selection can drive niche divergence while simultaneously preserving population-level functional diversity and generating variants highly adapted to specific landscape ecological niches. From a broader ecological and breeding perspective, future directional breeding of *Malus* should not be confined to a single extreme architectural type (e.g., over-intensifying weeping forms). Instead, it should strategically integrate high-functional-richness types (ST/UT) to enhance the population’s comprehensive resilience against multiple stresses, including climate change, light competition, and pests/diseases. Concurrently, the high functional divergence of WT should be fully exploited to develop cultivars specialized for extreme landscapes (e.g., vertical urban greening or waterfront weeping features). Such an approach would achieve synergistic optimization of functional diversity conservation, ecological service enhancement, and ornamental value.

### 3.3. Multiscale Allometric Scaling Differences Among Different Architectural Types in Malus and Their Resource Allocation Strategies

In this study, Standardized Major Axis (SMA) regression was applied across three hierarchical scales—whole-tree, branch, and leaf—to reveal significantly divergent allometric scaling patterns among the three architectural types of *Malus* germplasm (Figure 4).

At the whole-tree level, the UT exhibited a negative allometric relationship between tree height and ground diameter (slope = −0.482), whereas the WT showed strong positive allometry (slope = 1.177). This partially aligns with the conserved height–diameter allometry reported by Hulshof et al. [23]; however, the negative slope in UT likely reflects a mature-stage height-maintenance strategy or structural constraints imposed by its narrow crown [46]. Physiologically, as trees mature, hydraulic limitations (longer water transport paths) and mechanical stability demands (increased wind loads) shift resource allocation from vertical extension to radial growth; Mäkelä and Valentine [47] further demonstrated that low crown ratios amplify the disproportionate response of diameter to height: once crown ratio stabilizes in mature trees, resources are preferentially allocated to radial growth rather than continued height extension, thereby balancing gravitational and wind loads.

At the branch level, all three types followed positive allometric scaling between shoot length and basal diameter with slopes greater than 1 (common slope test, *p* = 0.327), consistent with Corner’s rule [24] and the classic trade-off between mechanical support and spatial expansion. Notably, WT displayed the highest intercept (*el* = 2.580), indicating greater radial investment per unit length to counteract the bending moments induced by its pendulous habit. This observation agrees with Brym and Ernest [48], who reported elevated intercepts in orchard trees under low-intensity or unpruned conditions, where branches must bear higher natural gravitational loads.

At the leaf level, the negative allometric relationship between petiole length and leaf area (slopes < 1) is consistent with the findings of Li et al. [26] on temperate woody species. Migicovsky et al. [49] further confirmed that leaf-shape allometric variation in apple has high heritability, supporting the observed phenotypic differentiation in the relationship between leaf shape index and internode length: negative allometry in UT versus positive allometry in both ST and WT.

Collectively, these multiscale differences illuminate the resource allocation logic underlying distinct tree architectures in *Malus*. WT prioritizes vertical height extension and radial reinforcement to maintain biomechanical stability of pendulous branches, with leaves tending toward elongated shapes adapted to the loose canopy’s light and thermal microenvironment. UT emphasizes mechanical compactness and light-capture optimization, featuring more orbicular, densely arranged leaves that maximize photosynthetic efficiency per unit branch length while minimizing self-shading. ST, in turn, exhibits superior spatial-filling efficiency, developing dense node production and leaf morphologies highly responsive to extension, thereby achieving synergistic advantages in horizontal expansion and light-resource capture.

Thus, this study provides the first cross-hierarchical quantitative demonstration in *Malus* of the multiscale resource trade-offs driven by tree architecture. It not only supplements ecological case studies on the structure–function co-evolution of perennial woody plants but also supplies a robust theoretical and empirical framework for developing early-stage, functional-trait-based selection models in directional breeding programs.

Accessions with high SSR and CSI can be prioritized for upright type breeding targeting narrow-canopy, high-light-efficiency cultivars suitable for urban spaces. Selection for weeping type cultivars should focus on high internode length (1YIL) and petiole length (PL) combined with elevated branch allometric intercepts to ensure biomechanical stability and light penetration in pendulous canopies. For spreading type breeding aiming at broad adaptability, genotypes exhibiting high FRic should be preferred due to their greater phenotypic plasticity and niche breadth. Moreover, multiscale allometric parameters may serve as early predictive proxies for mature tree architecture, enabling non-destructive seedling-stage selection and shortening breeding cycles when integrated with genomic tools.

### 3.4. Limitations and Future Directions

Despite the robustness of our analyses, several limitations should be acknowledged. First, the unbalanced sample sizes across architectural types (WT: n = 20; UT: n = 40; ST: n = 146) may lead to slight underestimation of functional richness (FRic) in the weeping type, although kernel density estimation and PCA-based metrics are relatively insensitive to sample imbalance. Second, the study focused on static two-dimensional functional space and did not include tiered crown analysis from above or quantitative shading factors, which could further elucidate physiological advantages and light interception dynamics. Third, while we discussed the potential role of the *LAZY* gene family in regulating gravitropic set-point angle (GSA), direct molecular or dynamic developmental data across ontogenetic stages were not integrated.

Future research should address these gaps by: (1) expanding the weeping-type germplasm collection to achieve more balanced sampling; (2) incorporating 3D terrestrial laser scanning and computational modeling to quantify tiered crown architecture, self-shading effects; (3) combining functional trait data with transcriptomic or CRISPR-based validation of key regulatory genes (e.g., *LAZY*, *WEEP*) to establish causal links between genotype, phenotype, and ecological performance. Such integrative approaches will further strengthen the theoretical foundation for functional trait-assisted breeding in ornamental woody plants; and (4) although the present study prioritizes quantitative functional trait data, it does not include photographic documentation, three-dimensional models of representative trees for each architectural type, or supplementary diagrams of the full crown systems. Future studies could incorporate such visual elements to further enhance the interpretability of architectural differences.

## 4. Materials and Methods

### 4.1. Experimental Site and Materials

A total of 206 *Malus* germplasm accessions, comprising 35 species, 94 North American cultivars, and 77 self-bred cultivars (Table 1; see the detailed list in Supplementary Table S1), were obtained from the National Repository of Crabapple Germplasm of China in Yangzhou (Jiangdu District, Yangzhou City, Jiangsu Province, China. 119°55′ E, 32°42′ N). To eliminate the potential confounding effects of different rootstocks on tree vigor and architectural phenotypes, all evaluated accessions were grafted onto uniform *Malus* hupehensis rootstocks. The experimental trees were of a consistent age (7–10 years old), with 10 replicate individuals per accession. All plants were cultivated in the same experimental plot at a spacing of 2 m × 3 m. Standardized field management practices, including uniform irrigation and fertilization, were applied, and no artificial pruning was performed to interfere with the natural tree architecture.

### 4.2. Architectural Phenotyping and Classification

This study aims to explore the underlying allometric scaling and functional tradeoffs among different architectural types in *Malus*. Accordingly, based on the phenotypic evaluation of primary branching patterns outlined in the *Guidelines for the Conduct of Tests for Distinctness, Uniformity and Stability—Ornamental Apple* (*Malus* Mill. except *Malus domestica* Borkh) (LY/T 3393-2024) [50], we classified the evaluated *Malus* accessions into three typical architectural types:

(1) Weeping type (WT): characterized by pendulous primary branches driven downward by gravity, forming an umbrella-shaped canopy.

(2) Upright type (UT): characterized by nearly vertical primary branches with extremely narrow crotch angles (typically <45°), forming a vertically oriented canopy.

(3) Spreading type (ST): characterized by obliquely upward or nearly horizontal primary branches with wider crotch angles (typically 45–90°), resulting in a laterally open canopy.

To ensure the purity of the subsequent comparative analyses, accessions exhibiting intermediate phenotypes or those severely affected by external environmental factors were excluded from this study.

### 4.3. Measurement of Basic Morphological Traits

**Figure 5:**
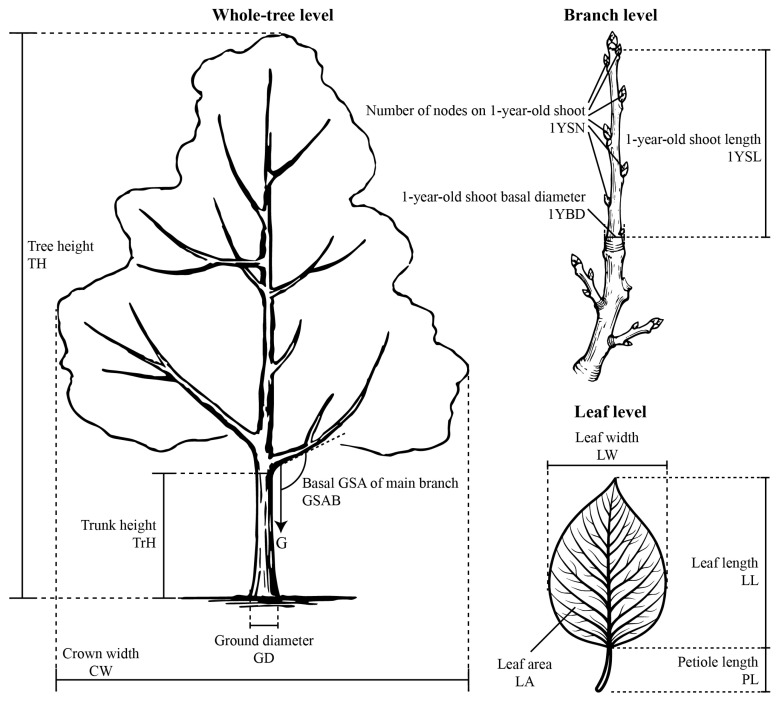
Schematic diagram of the measurement of 12 basic morphological traits across three structural levels (whole-tree, branch, and leaf) in *Malus*.

The observation of whole-tree and branch traits was conducted from November to December in both 2024 and 2025, while leaf measurements were performed from June to July during the same years. Following the principles of representativeness and consistency, all measurements were taken on sunny mornings. Healthy, pest-free individuals were selected, and branches as well as healthy, mature leaves located in the middle-to-upper canopy on the sunlit side were chosen for measurement. For each accession, 10 representative individuals were selected for the measurement of whole-tree traits, while 30 representative samples were evaluated for branch and leaf traits (i.e., 3 randomly selected branches or leaves from each of the 10 individuals). All measurements were averaged to yield a single representative value per trait for each accession. A total of 12 basic phenotypic traits were obtained across three structural levels:

(1) Whole-tree level: Tree height (TH, cm), ground diameter (GD, cm), crown width (CW, cm), and trunk height (TrH, cm) were measured (Figure 5).

(2) Branch level: Measurements included 1-year-old shoot length (1YSL, cm), number of nodes on the 1-year-old shoot (1YSN), 1-year-old shoot basal diameter (1YBD, cm), and the basal gravitropic set-point angle of the main branch (GSAB, °). Here, the main branch was defined as a permanent scaffold branch attached to the central trunk, and its base was designated at a point 10% of the total branch length from the trunk junction. The GSAB was measured using a protractor equipped with a plumb bob, with the gravity vector strictly defined as 0° [51] (Figure 5).

(3) Leaf level: Leaf length (LL, cm), leaf width (LW, cm), leaf area (LA, cm^2^), and petiole length (PL, cm) were measured (Figure 5).

A schematic diagram illustrating the measurement of these basic morphological traits is provided in Figure 5.

### 4.4. Derivation of Core Functional Traits

Although absolute structural dimensions (e.g., tree height and shoot length) are suitable for fitting allometric scaling models, they are often strongly influenced by absolute tree size (i.e., the size effect). To eliminate size effects and accurately capture the underlying resource allocation and ecological strategies of the plants, we selected and derived 9 core functional traits with explicit biomechanical and ecological significance based on the 12 basic measurements mentioned above. These traits were used for the subsequent construction of the functional trait space. The 9 core functional traits comprise 4 directly measured key structural indicators and 5 derived proportional traits, corresponding to three major functional categories: macro-architecture, branch biomechanics, and leaf economics. The specific calculation formulas and ecological significance of these traits are detailed in Table 2.

### 4.5. Statistical Analysis

All data processing, statistical analyses, and graphical visualizations were performed in the R environment version 4.3.1 [52]. Although the sample size for the weeping type (n = 20) is smaller than that for the spreading type (n = 146), kernel density estimation and PCA-based functional diversity metrics are robust to sample size imbalances [44].

(1) Functional Trait Space and Principal Component Analysis (PCA): Prior to the PCA, the nine core functional traits were Z-score standardized. This standardization was consistently applied across all subsequent multivariate analyses (including the calculation of functional diversity indices) to eliminate the influence of scale differences among traits on the construction of the functional space. The first two principal component axes were utilized to construct a two-dimensional trait space, and trait loadings were extracted to identify the primary drivers of variation within the functional trait space.

(2) Functional diversity indices: Based on the multidimensional coordinates derived from the PCA, we calculated functional richness (FRic, representing the absolute volume occupied within the functional space) and functional divergence (FDiv, indicating the degree of trait dispersion toward the margins of the trait space) for the three architectural types. Furthermore, the pairwise functional space overlap (FOve) among the groups was quantified.

(3) Allometric scaling analysis: To assess differences in resource allocation rates across traits, standardized major axis (SMA) regression was applied to the log10-transformed bivariate relationships (${\mathrm{Log}}_{10}Y=\log_{10}a+b\log_{10}X$). A common slope test was executed utilizing the ‘smatr’ (version 3.4-8) [53] package.

(4) Data visualization: High-quality statistical graphics and multi-panel layouts were generated and assembled primarily using the ‘ggplot2’ (version 4.0.3) [54], ‘ggExtra’ (version 0.11.0) [55], and ‘cowplot’ (version 1.2.0) [56] packages.

## 5. Conclusions

Based on multidimensional functional trait space and multiscale allometric scaling analyses, this study systematically elucidates the differentiated strategies in structural development and resource allocation among various architectural types of *Malus*. The results demonstrate that tree architecture is synergistically driven by macro-architectural, branch biomechanical, and leaf economic traits, with different types exhibiting significant divergence within the functional space. Notably, the WT occupies a highly specialized niche, whereas the UT and ST types possess broader potential for ecological adaptation. Furthermore, the multiscale allometric scaling relationships clarify the trade-off mechanisms among mechanical stability, spatial expansion, and light resource utilization across the different architectures. From the perspective of functional ecology, this research deepens our understanding of the coordinated evolution between structure and function in woody plants, providing a solid theoretical basis for the directional breeding of *Malus* architectures and functional trait-assisted selection.

## Figures and Tables

**Table 1:** Distribution of *Malus* germplasm accessions by origin and architectural type.

Origin	Weeping Type (WT)	Upright Type (UT)	Spreading Type (ST)	Total
Species	0	20	15	35
North American cultivars	7	15	72	94
Self-bred cultivars	13	5	59	77
Total	20	40	146	206

**Table 2:** Description, derivation formulas, and ecological significance of the 9 core functional traits used to construct the multidimensional functional space.

Functional Category	Functional Trait	Abbrev.	Derivation/Unit	Ecological Significance
Macro- architecture	Stem slenderness ratio	SSR	TH/GD	Reflects the biomechanical trade-off between vertical growth (light competition) and mechanical stability against wind throw or buckling [23].
	Crown shape index	CSI	TH/CW	Indicates the spatial strategy of the canopy (vertical vs. lateral expansion) and light interception efficiency [2].
	Crown ratio	CR	(TH-TrH)/TH	Represents the proportion of the vertical space occupied by the active photosynthetic canopy [47].
Branch biomechanics	Internode length of 1-year-old shoot	1YIL	1YSL/1YSN	Reflects the extension efficiency of shoot growth and the spatial compactness of foliage [28].
	1-year-old shoot basal diameter	1YBD	Measured (cm)	Represents the structural investment, mechanical support capability, and hydraulic transport potential of individual branches [11].
	Basal GSA of main branch	GSAB	Measured (°)	Determines the spatial trajectory of primary branches, heavily influencing the overarching crown architecture and gravity-induced mechanical stress [51].
Leaf economics	Leaf shape index	LSI	LL/LW	Relates to boundary layer resistance, thermal dissipation, and the avoidance of self-shading within the canopy microenvironment [27].
	Leaf area	LA	Measured (cm^2^)	A key indicator of the leaf economics spectrum, determining the light interception surface area and carbon construction cost per leaf [10].
	Petiole length	PL	Measured (cm)	Reflects the physical displacement strategy to optimize light capture and minimize mutual shading among adjacent leaves [14].

## Data Availability

The original contributions presented in this study are included in the article/Supplementary Materials. Further inquiries can be directed to the corresponding author. The detailed list of 206 germplasm accessions is provided in Supplementary Table S1.

## Supplementary Materials

The following supporting information can be downloaded at: https://www.mdpi.com/article/10.3390/plants15091347/s1, Table S1: Detailed list of 206 evaluated *Malus* germplasm accessions.
