# Supplementary material for: Functional Trait Space and Multiscale Allometric Scaling of Different Architectural Types in Malus
Source: Plants (Basel). 2026 Apr 28;15(9):1347. doi: 10.3390/plants15091347 (PMC13164757; doi:10.3390/plants15091347)
Supplement: Supplementary file 1 [file plants-15-01347-s001.zip › plants-4246716-supplementary.pdf]

Supplementary Materials:

Table S1. Detailed list of 206 evaluated *Malus* germplasm accessions.

No.	Name	Origin	Architectural type	No.	Name	Origin	Architectural type
1	*Malus angustifolia*	Species	ST	104	*M.* 'Royal Beauty'	NA cultivars	WT
2	*M. asiatica*	Species	UT	105	*M.* 'Royal Gem'	NA cultivars	ST
3	*M. asiatica* var. *rinki*	Species	UT	106	*M.* 'Royal Raindrop'	NA cultivars	ST
4	*M. baccata*	Species	UT	107	*M.* 'Royalty'	NA cultivars	ST
5	*M. baccata* var. *Mandshurica*	Species	UT	108	*M.* 'Selkirk'	NA cultivars	UT
6	*M. coronaria*	Species	UT	109	*M.* 'Sentinel'	NA cultivars	ST
7	*M. domestica*	Species	ST	110	*M.* 'Shelley'	NA cultivars	ST
8	*M. doumeri*	Species	ST	111	*M.* 'Show Girl'	NA cultivars	UT
9	*M.* × *floribunda*	Species	ST	112	*M.* 'Show Time'	NA cultivars	ST
10	*M. fusca*	Species	ST	113	*M.* 'Snowdrift'	NA cultivars	ST
11	*M. halliana*	Species	ST	114	*M.* 'Sparkler'	NA cultivars	ST
12	*M. hupehensis*	Species	ST	115	*M.* 'Spring Glory'	NA cultivars	ST
13	*M. ioensis*	Species	ST	116	*M.* 'Spring Sensation'	NA cultivars	ST
14	*M. kirghisorum*	Species	UT	117	*M.* 'Spring Snow'	NA cultivars	ST
15	*M.* × *micromalus*	Species	UT	118	*M.* 'Strawberry Jelly'	NA cultivars	ST
16	*M. orientalis*	Species	ST	119	*M.* 'Sugar Tyme'	NA cultivars	ST
17	*M.* × *platycarpa*	Species	UT	120	*M.* 'Sweet Sugartyme'	NA cultivars	ST
18	*M. prattii*	Species	UT	121	*M.* 'Thunderchild'	NA cultivars	UT
19	*M. prunifolia*	Species	UT	122	*M.* 'Vans Eseltine'	NA cultivars	ST
20	*M.* × *robusta*	Species	UT	123	*M.* 'Velvet Pillar'	NA	ST

						cultivars	
21	*M. rockii*	Species	UT	124	*M.* 'Weeping Madonna'	NA cultivars	ST
22	*M. sargentii*	Species	ST	125	*M.* 'White Cascade'	NA cultivars	WT
23	*M. sieversii*	Species	UT	126	*M.* 'Winter Gold'	NA cultivars	ST
24	*M. sieversii* f. *Niedzwetzkyana*	Species	UT	127	*M.* 'Winter Red'	NA cultivars	ST
25	*M. sikkimensis*	Species	UT	128	*M.* 'Yellow Jade'	NA cultivars	UT
26	*M. spectabilis*	Species	UT	129	*M.* × *zumi* 'Calocarpa'	NA cultivars	ST
27	*M. sylvestris*	Species	ST	130	*M.* 'Ajiao'	Self-bred	ST
28	*M. toringo*	Species	ST	131	*M.* 'Anni'	Self-bred	UT
29	*M. toringoides*	Species	ST	132	*M.* 'Bawang Bieji'	Self-bred	WT
30	*M. transitoria*	Species	ST	133	*M.* 'Beiguo Zhichun'	Self-bred	ST
31	*M. tschonoskii*	Species	UT	134	*M.* 'Caiyun Zhuiyue'	Self-bred	WT
32	*M. turkmenorum*	Species	UT	135	*M.* 'Chahua Nv'	Self-bred	ST
33	*M. xiaojinensis*	Species	ST	136	*M.* 'Chanjuan'	Self-bred	ST
34	*M. xinjinensis*	Species	UT	137	*M.* 'Chudong'	Self-bred	ST
35	*M. yunnanensis*	Species	UT	138	*M.* 'Chuqiu'	Self-bred	ST
36	*M.* 'Abundance'	NA cultivars	ST	139	*M.* 'Dabai Kongque'	Self-bred	ST
37	*M.* 'Adams'	NA cultivars	ST	140	*M.* 'Denghuo Lanshan'	Self-bred	ST
38	*M.* 'Addirondack'	NA cultivars	UT	141	*M.* 'Dianjiang Chun'	Self-bred	ST
39	*M.* 'Almey'	NA cultivars	UT	142	*M.* 'Dongri Lianqing'	Self-bred	ST
40	*M.* 'America Salute'	NA cultivars	UT	143	*M.* 'Duojiao'	Self-bred	UT
41	*M.* 'American Spirit'	NA cultivars	UT	144	*M.* 'Fenba Lei'	Self-bred	ST
42	*M.* 'Ballet'	NA cultivars	UT	145	*M.* 'Fendai'	Self-bred	ST
43	*M.* 'Ballet Red'	NA cultivars	UT	146	*M.* 'Fenhong Nichang'	Self-bred	ST
44	*M.* 'Big Red'	NA cultivars	ST	147	*M.* 'Gaoshan Liushui'	Self-bred	WT
45	*M.* 'Black Jade'	NA cultivars	WT	148	*M.* 'Hongkong Que'	Self-bred	ST

46	*M.* 'Brandywine'	NA cultivars	ST	149	*M.* 'Honglou'	Self-bred	ST
47	*M.* 'Bride'	NA cultivars	ST	150	*M.* 'Hongluo'	Self-bred	ST
48	*M.* 'Butterball'	NA cultivars	ST	151	*M.* 'Hongqi Bing'	Self-bred	ST
49	*M.* 'Candymint'	NA cultivars	ST	152	*M.* 'Hongting'	Self-bred	ST
50	*M.* 'Cardinal'	NA cultivars	ST	153	*M.* 'Hongwang Zi'	Self-bred	ST
51	*M.* 'Centurion'	NA cultivars	ST	154	*M.* 'Huamu Lan'	Self-bred	ST
52	*M.* 'Chestnut'	NA cultivars	ST	155	*M.* 'Huangguo Shu'	Self-bred	WT
53	*M.* 'Cinderella'	NA cultivars	ST	156	*M.* 'Huangjin Haian'	Self-bred	ST
54	*M.* 'Coccinella'	NA cultivars	ST	157	*M.* 'Huanxi Sha'	Self-bred	ST
55	*M.* 'David'	NA cultivars	ST	158	*M.* 'Hudie Quan'	Self-bred	WT
56	*M.* 'Dolgo'	NA cultivars	ST	159	*M.* 'Jiangnan An'	Self-bred	ST
57	*M.* 'Donald Wyman'	NA cultivars	ST	160	*M.* 'Jiangnan Chunxiao'	Self-bred	ST
58	*M.* 'Eleyi'	NA cultivars	ST	161	*M.* 'Jiangnan Tianyuan'	Self-bred	WT
59	*M.* 'Everest'	NA cultivars	ST	162	*M.* 'Jiangtian Yise'	Self-bred	ST
60	*M.* 'Fairytail Gold'	NA cultivars	UT	163	*M.* 'Jingang Wan'	Self-bred	ST
61	*M.* 'Firebird'	NA cultivars	ST	164	*M.* 'Jinqiu'	Self-bred	WT
62	*M.* 'Flame'	NA cultivars	ST	165	*M.* 'Jintong'	Self-bred	ST
63	*M.* 'Golden Hornet'	NA cultivars	ST	166	*M.* 'Jinxin Guo'	Self-bred	ST
64	*M.* 'Golden Raindrop'	NA cultivars	ST	167	*M.* 'Jinxiu Jiangnan'	Self-bred	ST
65	*M.* 'Gorgeous'	NA cultivars	ST	168	*M.* 'Jinyue Liang'	Self-bred	WT
66	*M.* 'Guard'	NA cultivars	ST	169	*M.* 'Jiwei Jiu'	Self-bred	UT
67	*M.* 'Harvest Gold'	NA cultivars	ST	170	*M.* 'Lanting'	Self-bred	UT

68	*M.* 'Hopa'	NA cultivars	ST	171	*M.* 'Lianjuan Xifeng'	Self-bred	WT
69	*M.* 'Hydrangea'	NA cultivars	ST	172	*M.* 'Linjiang Xian'	Self-bred	ST
70	*M.* 'Indian Magic'	NA cultivars	ST	173	*M.* 'Lvpao Pao'	Self-bred	ST
71	*M.* 'Indian Summer'	NA cultivars	ST	174	*M.* 'Orange Dream'	Self-bred	ST
72	*M.* 'John Downie'	NA cultivars	ST	175	*M.* 'Qianceng Jin'	Self-bred	ST
73	*M.* 'Kelsey'	NA cultivars	UT	176	*M.* 'Qianfan Jingxiu'	Self-bred	ST
74	*M.* 'King Arthur'	NA cultivars	ST	177	*M.* 'Qingliang Yixia'	Self-bred	ST
75	*M.* 'Klehm's Improved Bechtel'	NA cultivars	ST	178	*M.* 'Qingyu An'	Self-bred	WT
76	*M.* 'Lancelot'	NA cultivars	ST	179	*M.* 'Qingzhi Manwu'	Self-bred	ST
77	*M.* 'Lemoinei'	NA cultivars	UT	180	*M.* 'Qinyuan Chun'	Self-bred	ST
78	*M.* 'Lisa'	NA cultivars	ST	181	*M.* 'Qiuhuo Yan'	Self-bred	ST
79	*M.* 'Liset'	NA cultivars	ST	182	*M.* 'Qiukong Que'	Self-bred	ST
80	*M.* 'Lollipop'	NA cultivars	ST	183	*M.* 'Qiuri Siyu'	Self-bred	ST
81	*M.* 'Louisa'	NA cultivars	WT	184	*M.* 'Qiuyun'	Self-bred	ST
82	*M.* 'Louisa Contort'	NA cultivars	WT	185	*M.* 'Shanhu Hua'	Self-bred	ST
83	*M.* 'Makamik'	NA cultivars	ST	186	*M.* 'Shanli Hong'	Self-bred	ST
84	*M.* 'Mary Potter'	NA cultivars	ST	187	*M.* 'Tiannv Sanhua'	Self-bred	WT
85	*M.* 'Mays Delight'	NA cultivars	ST	188	*M.* 'Tiaose Ban'	Self-bred	ST
86	*M.* 'Molten Lava'	NA cultivars	ST	189	*M.* 'Tingting Yuli'	Self-bred	ST
87	*M.* 'Neville Copeman'	NA cultivars	ST	190	*M.* 'Wanyan'	Self-bred	ST
88	*M.* 'Painted Scroll'	NA cultivars	UT	191	*M.* 'Xiangfei'	Self-bred	UT
89	*M.* 'Perfect Purple'	NA cultivars	ST	192	*M.* 'Xiangya'	Self-bred	ST

90	*M.* 'Pink Prince'	NA cultivars	WT	193	*M.* 'Xiangye'	Self-bred	ST
91	*M.* 'Pink Spires'	NA cultivars	ST	194	*M.* 'Xiaoer Lang'	Self-bred	ST
92	*M.* 'Praire Rose'	NA cultivars	ST	195	*M.* 'Xiaowei'	Self-bred	ST
93	*M.* 'Prairifire'	NA cultivars	ST	196	*M.* 'Xingxing Suo'	Self-bred	ST
94	*M.* 'Professor Sprenger'	NA cultivars	ST	197	*M.* 'Xintian You'	Self-bred	WT
95	*M.* 'Profusion'	NA cultivars	ST	198	*M.* 'Yipin Hong'	Self-bred	ST
96	*M.* 'Purple Prince'	NA cultivars	UT	199	*M.* 'Yutang Chun'	Self-bred	WT
97	*M.* 'Radiant'	NA cultivars	ST	200	*M.* 'Zihan'	Self-bred	ST
98	*M.* 'Red Baron'	NA cultivars	ST	201	*M.* 'Ziou'	Self-bred	ST
99	*M.* 'Red Jewel'	NA cultivars	ST	202	*M.* 'Zise Meihuo'	Self-bred	ST
100	*M.* 'Red Sentinel'	NA cultivars	ST	203	*M.* 'Zitang'	Self-bred	ST
101	*M.* 'Red Splendor'	NA cultivars	ST	204	*M.* 'Ziwei Xing'	Self-bred	ST
102	*M.* 'Robinson'	NA cultivars	ST	205	*M.* 'Ziyu Zhui'	Self-bred	ST
103	*M.* 'Roger' s Selection'	NA cultivars	WT	206	*M.* 'Ziyun Xuan'	Self-bred	ST

Note: Morphotype corresponds to the architectural type defined in this study (WT, weeping type; UT, upright type; ST, spreading type). NA, North American.
